# P300 in workers exposed to occupational noise

**DOI:** 10.5935/1808-8694.20120042

**Published:** 2015-10-20

**Authors:** Camila Gonçalves Polo Massa, Camila Maia Rabelo, Renata Rodrigues Moreira, Carla Gentile Matas, Eliane Schochat, Alessandra Giannella Samelli

**Affiliations:** a Specialist in Audiology (Speech and Hearing Therapist); b PhD in Communicaton Sciences, FMUSP (Speech and Hearing Therapist in the Department of Physiotherapy, Speech and Hearing Therapy, and Occupatonal Therapy at FMUSP); c PhD in Clinical Emergencies (Speech and Hearing Therapist at the USP University Hospital); d Associate Professor in the Department of Physiotherapy, Speech and Hearing Therapy, and Occupatonal Therapy at FMUSP (Associate Professor in the Department of Physiotherapy, Speech and Hearing Therapy, and Occupatonal Therapy at FMUSP); e PhD in Sciences, FMUSP (Professor in the Department of Physiotherapy, Speech and Hearing Therapy, and Occupatonal Therapy at the Medical School of the University of São Paulo)

**Keywords:** electrophysiology, event-related potentials, p300, speech perception

## Abstract

The harm upon the central auditory pathways of workers exposed to occupational noise has been scarcely studied.

**Objective:**

To assess the central auditory pathways by testing the long latency auditory evoked potentials (P300) of individuals exposed to occupational noise and controls.

**Method:**

This prospective study enrolled 25 individuals with normal hearing thresholds. The subjects were divided into two groups: individuals exposed to occupational noise (13 subjects; case group) and individuals not exposed to occupational noise (12 subjects; control group). The P300 test was used with verbal and non-verbal stimuli.

**Results:**

No statistically significant differences were found between ears for any of the stimuli or between groups. The groups had no statistically significant difference for verbal or non-verbal stimuli. Case group subjects had longer latencies than controls. In qualitative analysis, a greater number of altered P300 test results for verbal and non-verbal stimuli was seen in the case group, despite the absence of statistically significant differences between case and control subjects.

**Conclusion:**

Individuals exposed to high sound pressure levels had longer P300 latencies in verbal and non-verbal stimuli when compared to controls.

## INTRODUCTION

According to the National Institute for Occupational Safety and Health (NIOSH)^1^, approximately 30 million American workers are exposed to potentially harmful sound pressure levels. Noise-induced hearing loss (NIHL) ranks second among the occupational diseases affecting American workers^2^, ^3^, ^4^, while exposure to noise is the second most important cause of sensorineural hearing loss after presbycusis^5^.

In addition to hearing loss, prolonged exposure to noise may produce cardiovascular, psychological, and respiratory alterations, sleep disorders, immune system dysfunctions, irritability, and fatigue^2^, ^6^, ^7^. Noise can also reduce on-the-job performance levels and increase the chance of occupational accidents^8^, ^9^.

Studies have shown that aside from the harm inflicted upon one's peripheral auditory system and overall health status, prolonged exposure to noise can also alter cortical processing and thus affect the speed, strength, and topography of central auditory responses, in addition to impairing one's discourse production, cognitive performance, and short term memory. Additionally, it may affect the discrimination of verbal and non-verbal queues in different ways. Such outcomes were observed through the analysis of long latency evoked auditory potentials and behavioral responses, suggesting that individuals exposed to noise present altered brain function lateralization in speech processing in silent conditions, even when they have no peripheral auditory deficit^6^, ^7^.

These findings indicate that the assessment of auditory thresholds through audiometry should be enough to the determine the functional integrity of the central auditory system of individuals exposed to noise^6^.

The damage produced by exposure to noise upon the central auditory pathways verified by long latency auditory evoked potentials has been scarcely studied. Given the relevance of this topic and the possibility of developing preventive strategies to protect the entire auditory system, this study aimed to assess the central auditory pathways by analyzing long latency evoked auditory potentials (P300) and presenting verbal and non-verbal stimuli to individuals with normal hearing exposed to high sound pressure levels.

## METHOD

This study was carried out as per the principles of the Declaration of Helsinki and was approved by the Ethics Committee of our institution (permits 925/09 and 0479/09). The participants of the study signed Informed Consent Terms.

Twenty-five healthy male individuals with normal hearing (thresholds of less than 25 dB as per Ordinance 19^10^) were enrolled in the study. The subjects were divided into two groups: one made up of individuals exposed to occupational noise (13 subjects; case group) and another containing subjects not exposed to occupational noise (12 individuals; control group). The mean age of control group subjects was approximately 35 years (SD = 13.76), while case group individuals had a mean age of 40 years (SD = 6.57). Mean group ages were not statistically different (*p* = 0.126).

Subjects in the case group had to be exposed to occupational noise for over five years at sound pressure levels above 85 dB, eight hours a day. The measurements of noise levels was made by the Occupational Health and Safety Division.

All subjects underwent thorough audiological examination and had their long and short latency auditory evoked potentials tested with verbal and non-verbal stimuli.

Before having short and long latency auditory evoked potentials tested, the subjects had their skin cleaned with abrasive paste and electrodes put in place with electrolytic paste and adhesive tape. Electrode impedance values of 5,000 ohms and under were considered.

Brainstem auditory evoked potential (BAEP) tests were carried out initially as part of the patient enrollment process. All subjects had to have normal BAEPs and verified brainstem integrity to avoid biased outcomes in the ensuing tests, once peripheral auditory system or brainstem dysfunction may alter P300 test results. The subjects were submitted to P300 testing only after morphology and absolute latencies for waves I, III, and V and interpeak intervals I-III, III-V, I-V, at 80 dBnNA, for clicks at a rate of 19.0 stimuli per second were confirmed to be normal.

During the P300 tests, the electrodes were placed on the vertex (Cz - reference), forehead (Fpz - ground), and left (A1) and right (A2) mastoid. Participants were asked to keep their eyes closed to avoid interference from eye motions and to count low-probability target stimuli out loud (20% of the total number of stimuli) as they randomly appeared amongst high-probability nontarget stimuli (80% of the total number of stimuli) - the oddball paradigm. One ear was assessed each time.

The equipment used in this test was Bio-logic's Auditory Evoked Potential (AEP) System connected to a Microboard workstation. A set of Bio-logic in-ear headphones was used to acquire P300 test data.

Non-verbal stimuli (tone burst with 30-ms plateau and 10-ms rise/fall times) were presented at 1,000 Hz (high-probability stimuli) and 2,000 Hz (low-probability stimuli); and verbal stimuli (syllables/ba/for high-probability sounds and/da/for low-probability sounds) at 75 dB nNA, at a rate of 1.1 stimuli per second; time of analysis: 800 ms; 1 Hz high pass filter and 15 Hz low pass filter; gain: 50000; sensitivity: 100 microvolts. Three hundred stimuli were used for each stimulus type. Only one record was produced for each side (ipsilateral) on each stimulus mode, and the reproduction of these waves was not recorded, as replication of the data collection could exhaust subjects and compromise the outcome of the assessment, as it is greatly dependent on sustained attention.

P300 was identified as a wave of positive polarity occurring approximately 300 ms after the stimulus, obtained after the tracing of low-probability stimuli was subtracted from the tracing related to high-probability stimuli. P300 latency was assessed, as this parameter is more reliable than amplitude^11^.

Statistical analysis was carried out using ANOVA - Analysis of Variance. This is a commonly used parametric technique in which mean values are compared in terms of their variances. Fisher's exact test was used in qualitative analysis. A level of significance of 0.05 (5%) was adopted in this study. This statistical approach is suitable for small samples, as is the case of this study.

## RESULTS

### Quantitative analysis - P300

#### Non-verbal stimuli

The P300 results for non-verbal stimuli showed no statistically significant differences between the right and left ears of either of the groups (Table 1).

**Table 1:** Mean values, standard deviations, and p-values of P300 wave (non-verbal stimuli) latencies (in milliseconds) of control and case group subjects, per ear.

P300 Non-verbal	Control Group		Case Group	
	RE	LE	RE	LE
Mean	313.86	328.09	340.65	340.65
SD	33.33	22.2	25.12	28.17
p-value	0.232		1.000	

RE: Right Ear; LE: Left Ear; SD: Standard Deviation.

Thus, in order to compare the groups, right and left ears were analyzed together in each group (Table 2). Statistically significant difference was observed between groups, with case group subjects presenting increased P300 latencies when compared to controls.

**Table 2:** Mean values, standard deviations, and p-values of P300 wave (non-verbal stimuli) latencies (in milliseconds) of control and case group subjects.

P300 non-verbal	Control Group	Case Group
Mean	320.97	340.65
SD	28.63	26.15
*p*-value	0.014*	

^*^ statistically significant difference (ANOVA).

#### Verbal stimuli

The P300 results for verbal stimuli showed no statistically significant differences between the right and left ears (Table 3).

**Table 3:** Mean values, standard deviations, and p-values of P300 wave (verbal stimuli) latencies (in milliseconds) of control and case group subjects, per ear.

P300 - verbal	Control Group		Case Group	
	RE	LE	RE	LE
Mean	356.02	341.88	362.27	369.08
SD	24.06	33.99	32.62	41.08
*p*-value	0.252		0.643	

RE: Right Ear; LE: Left Ear; SD: Standard Deviation.

Thus, in order to compare the groups, right and left ears were analyzed together in each group (Table 4). Statistically significant difference was observed between groups, with case group subjects presenting increased P300 latencies with verbal stimuli when compared to controls.

**Table 4:** Mean values, standard deviations, and *p*-values of P300 wave (verbal stimuli) latencies (in milliseconds) of control and case group subjects.

P300 - verbal	Control Group	Case Group
Mean	346.45	365.67
SD	29.97	36.51
*p*-value	0.048*	

^*^ statistically signifcant difference. SD: Standard Deviation.

### Qualitative analysis - P300

No statistically significant differences were found in the distribution of normal and altered test results when both groups were compared, for P300 tests with non-verbal (Table 5) and verbal (Table 6) stimuli. However, for both stimulus modes the case group had more altered test results than the control group, with verbal stimuli posting a more significant difference (mean ± 1 SD).

**Table 5:** Distribution of normal and altered P300 waves with non-verbal stimuli among case and control groups considering the reference values proposed by McPherson^12^.

	Altered	Normal	Total
Case Group	2	11	13
Control Group	0	12	12
Total	2	23	25

*p*-value = 0. 52.

**Table 6:** Distribution of normal and altered P300 waves with verbal stimuli among case and control groups considering the reference values proposed by Massa^13^.

	Altered	Normal	Total
Case Group	7	6	13
Control Group	3	9	12
Total	10	15	25

*p*-value = 0.172.

## DISCUSSION

Damage to the peripheral auditory pathways of noise-exposed workers has been documented through pure-tone audiometry. Nonetheless, the damages caused by noise to central auditory processing has been scarcely studied in this population. Some studies have shown that prolonged exposure to noise may also alter cortical processing, discourse production, cognitive performance, and short term memory.

Alterations in the brain function lateralization of speech processing in silent conditions have been found in individuals exposed to noise with normal peripheral hearing^6^, ^7^. With that in mind, this study attempted to assess the central auditory pathways by the P300 waves of subjects with normal hearing exposed to occupational noise.

There were no statistically significant differences between the right and left ears in each group for non-verbal or verbal stimuli. This finding is in agreement with reports in the literature which failed to find statistically significant differences between ears for P300 latency assessed through click stimuli in adults with normal hearing^14^. There are no published comparisons between ears in P300 testing with verbal stimuli. A few studies have looked into verbal stimuli, but none mention differences between ears or brain hemispheres^13^.

However, as the groups were compared for non-verbal and verbal stimuli, latencies were statistically increased in the case group.

Considering the distribution of normal and altered P300 waves as per the classification scheme proposed by McPherson^12^, no statistically significant differences were found between groups. However, still according to this classification, two subjects had altered P300 test results in the case group.

When taking the reference mean P300 latency values published by Massa^13^, no statistically significant differences were found for the distribution of normal and altered test results in the groups. However, the case group had a greater number of altered test results (7) when compared to the control group (3).

Studies have shown that individuals with brain injury may have delayed or altered P300 waves, despite their ability to correctly perform the task of counting low-probability stimuli, indicating that there is some impact from neural plasticity in these cases^15^. Based on this assumption, one may infer that individuals exposed to noise have a greater likelihood of having auditory processing disorders, due to their increased P300 latencies and greater number of alterations as seen in this study.

Considering the P300 test results with nonverbal and verbal stimuli, greater mean latencies were seen in the case group. This fact may suggest that case group subjects process acoustic stimuli more slowly than controls; thus, one may infer that exposure to occupational noise changes the way acoustic stimuli are processed - particularly speech - and possibly alters central auditory processing in subjects submitted to prolonged exposure to occupational noise^6^, ^7^.

Even though quantitative and qualitative analyses have revealed different results, and despite the failure in finding statistically significant differences in qualitative analysis, quantitative analysis showed a marked difference between the two groups. It is recommended that further studies be carried out with larger sample sizes to allow the investigation of the differences arising from qualitative analysis.

Differences in auditory central processing were observed between individuals exposed to occupational noise and controls.

Recent studies have shown that the introduction of noise during the acquisition of evoked potentials may negatively affect the amplitude and/or latency of the waves of the potentials of short, medium, and/or long latency^16^, ^17^, ^18^. The effect noise has on auditory evoked potentials may be mediated by the efferent auditory system^19^. Based on this statement and considering the results found in this study, one may suppose that the increased latencies seen in case group subjects could be the consequence of auditory nervous system plasticity from prolonged exposure to occupational noise; the ensuing auditory processing disorder would be visualized by increased P300 latencies for verbal and non-verbal stimuli. Therefore, future studies could use noise during the acquisition of auditory evoked potentials while assessing this population, to thus verify how the processing of acoustic stimuli takes place in groups exposed to noise.

## CONCLUSION

Individuals exposed to noise had statistically greater mean P300 latencies in tests done with verbal and non-verbal stimuli when compared to controls.
